# “Thermal Peroxidation” of Dietary Pentapeptides Yields N-Terminal 1,2-Dicarbonyls

**DOI:** 10.3389/fnut.2021.663233

**Published:** 2021-07-22

**Authors:** Maria Bikaki, Nikolai Kuhnert

**Affiliations:** Department of Life Sciences and Chemistry, Jacobs University Bremen, Bremen, Germany

**Keywords:** peptides, mass spec, thermal processing, oxidation, food, dicarbonyls

## Abstract

In this contribution we investigate the thermal degradation of dietary-relevant pentapeptides. Most unsaturated lipids degrade by the well-known peroxidation mechanism. Here we show a degradation mechanism of peptides analogous to lipid peroxidation, forming a series of novel degradation products with possible toxicological relevance. At elevated temperatures above 180°C, pentapeptides with an N-terminal phenylalanine moiety react *via* a debenzylation to form 1,2-dicabonyl compounds, replacing the N-terminal primary amine. We propose a radical-based reaction mechanism that leads *via* a common peroxoaminal intermediate to two distinct types of reaction products with a terminal α-1,2 diamide or an α-amide-aldehyde functionality.

## Introduction

The majority of protein rich food is processed by thermal treatment prior to human consumption. Thermal processing of food is a unique human activity and has been carried out, according to archeological evidence, by our ancestors for at least 1 million years (1). Thermal treatment includes a wide range of temperature and time regimes commonly referred to as cooking, baking, frying, steaming, roasting, and others. As multifunctional molecules embedded in a complex matrix, composed from a myriad of further constituents, proteins are thermally labile, changing conformation at lower temperatures (2) and undergoing a series of chemical transformations above 150°C, significantly altering the chemical composition of the food (3, 4). The most investigated of these transformation is the Maillard reaction of amino acids with sugars producing, *via* a series of intermediates, among small aroma active molecules, such as Strecker aldehydes or pyrazines, as well as ill-defined pigmented polymers, referred to as melanoidines (5–7).

Information available on the nature of thermally induced reaction products of proteins is rather scarce due to the complexity of the system. Even single amino acids have been found to form hundreds of products in the presence of glucose, depending on the nature of their side chain (8). Selected examples of structurally characterized degradation products of proteins include simple inorganic and organic compounds (CO_2_, H_2_O, NH_3_, and CO), with a variety of volatile organic compounds (amines, nitriles, amides, hydrocarbons, etc.) and lots of less volatile organic compounds such as diketopiperazines, lactams, and hydantoins.

When considering the detrimental or beneficial health effects and dietary impact of a given food, not only intact proteins in the raw material but all processing-induced products should be considered (7). Compounds obtained from thermal treatment of proteins and amino acids with well-studied detrimental effects on human health include polyaromatic heterocycles and acrylamide (9). A recent ILSI expert group study has highlighted the importance and unfortunate lack of information on food processing parameters in epidemiological studies (7). In particular, a series of recent epidemiological studies have highlighted, though still very controversial, an adverse effect of a high protein diet on human health with possible links to stroke and cardiovascular mortality (10–12). These studies pointed out that effects strongly depended on the nature of the protein source with dairy and vegetable proteins correlating to less adverse effect compared to meat proteins. This observation well illustrates the point of processing parameters since dairy proteins are mostly processed for pasteurization at temperatures below 100°C, most vegetables are consumed unprocessed or cooked at 100°C, whereas meat is typically subjected to higher temperatures.

However, in parallel to embarking on processing effects of protein rich diets on human health, an improved understanding of the chemistry underlying thermally-treated proteins and peptides is urgently required. A scientific approach for simplification of model system is the study of thermally-treated short peptides. Short peptides are ubiquitous in many foods, particularly if fermented or treated with proteolytic enzymes, and therefore are of dietary relevance (13, 14). Reactions that have been reported on thermally-treated proteins and peptides include breakage of amide bonds by residual water, intramolecular cleavage or diketopiperazine formation, dehydration and other elimination reactions, decarboxylation, oxidation of selected side chains including cysteine, methionine, and tryptophan, and cross-linking (4, 15).

We have recently reported two novel types of reaction products identified by tandem MS, which were found to be formed during heat treatment of dietary relevant pentapeptides. In this contribution, we would like to extend this work, reporting on a novel reaction type induced by heterolytic cleavage of benzyl moieties, which to our knowledge does not even haven an analogue in synthetic organic chemistry. The resulting dicarbonyl products are reminiscent of α-dicarbonyls, which have been intensely discussed and investigated in the field of so-called Advanced Glycation end Products (AGEs) and prompt speculation on possible health implications, demanding further investigations (16).

## Materials and Methods

### Chemicals and Reagents

Custom pentapeptides used in this study were synthesized from Synpeptide Co., Ltd., Shanghai, China and were used without further purification. Purity was assessed prior to the experiment using the HPLC-ESI-MS method described below and found to be >95% in all cases. All reagents used in this study were HPLC grade and they were purchased from Sigma-Aldrich (Germany). Milli-Q water (18.2 MΩ·cm at 25°C) was used throughout all experiments.

### Sample Preparation

Pentapeptides were heated in a laboratory oven at 220°C for 10 min. The solid peptide standards (50 μg each) were placed in an open 2 mL glass vial, heated, and then cooled down at room temperature. The non-volatile thermal degraded products were collected by extraction from inside the glass tube with milli-Q water (50 μL). 1:10 dilutions of the stock samples were directly used for HPLC-MS measurements. Three pentapeptides were heated and analyzed in triplicate to ensure reproducibility. Direct infusions on an Ion Trap mass spectrometer were performed when targeted fragmentation was required.

### UHPLC-ESI-Q-TOF-MS

HPLC experiments were performed on an Agilent 1260 HPLC system using a Peptide 2.7 μm C18 column (2.1 × 250 mm, 2.7 μm particle size) along with the recommended guard column. The sample injection volume was 3 μL. The binary solvent system used consisted of Milli-Q water (Solvent A) and acetonitrile (Solvent B), both containing 0.1% formic acid. The solvent's flow rate was kept constant at 0.250 mL/min and the column temperature was set at 25°C. The gradient profile used was: starting with 5% B, increasing to 100% B in 80 min, followed by washing with 100% B until 90 min, and decreasing to 5% B until 95 min. The column was re-equilibrated for the next measurement at 5% B for 20 more min.

The effluent HPLC system was connected to an Impact HD ultra-high resolution ESI-Q-TOF mass spectrometer (Bruker Daltonics, Bremen, Germany) coupled to an electrospray ionization source (nebulizer pressure of 2 bars, dry gas flow rate of 8 L/min, and dry gas temperature of 200°C). All data were acquired in both negative- and positive- ion mode. In this work only results in positive ion mode are presented. Both full scan spectra and MS/MS datasets were recorded. The TOF analyzer was calibrated with a 0.1 M sodium formate solution before each chromatographic run. Mass calibration was carried out in the enhanced quadratic mode, resulting in a mass accuracy of 0.8–1.0 ppm.

Charge de-convolution of HPLC-TOF-MS data was performed using DataAnalysis 4.2 (Bruker, Germany). All results presented in this study were analyzed manually using both tandem MS and high-resolution mass spectrometric data. When required, further manual targeted fragmentation of higher order MS^3^ and MS^4^ was performed with an ion-trap mass spectrometer fitted with an ESI source (HCT-Ultra, Bruker Daltonics, Bremen, Germany) in positive ion mode using identical chromatographic conditions as described above. Direct infusions were carried out in the positive ion mode using a target mass list with a Syringe pump infusing the stock solution used for HPLC injections at a flow rate of 180 μL/h.

## Results and Discussion

In order to understand the chemical behavior during thermal processing of food peptides, fifteen selected custom pentapeptides (Supplementary Table 1) with systematically varying amino acid sequences were subjected to thermal processing and subsequent LC-MS characterization using an ESI-Q-TOF MS instrument. This series constitutes a significant expansion on pentapeptides investigated previously (3).

Most pentapeptides selected carried a C-terminal tryptophan moiety and an N-terminal phenylalanine moiety to assist in compound identification by LC-UV analysis due to characteristic chromophores indicating the formation of C- or N-terminal breakdown products. Moreover, natural proteolytic enzymes with *exo*-peptidase activity have a tendency to cease degradation at aromatic amino acid residues (17), hence accumulating such peptides in typical fermented foods such as cocoa, dairy, or soy, adding particular relevance to these sequences from a dietary composition perspective. Additionally the peptides selected bear sequences already reported in dietary materials (3). In this contribution we use one letter abbreviations for amino acids, meaning that H_2_N-Phe-Ala-Lys-Ala-Trp-COOH is designated as FAKAW.

Mimicking conditions to those in real food systems, samples were heated at 180°C or at 220°C for 10 min in solid form in a laboratory oven under atmospheric conditions. The LC-MS signals of thermal degradation products were largely identical in both cases, however, at the lower temperature only 10% reaction yield was observed, if comparing product peak areas to starting material peak area. At the higher temperature the reaction yield was estimated at 40–80%, allowing acquisition of good quality tandem MS data for all major degradation products. Hence, all further work was continued at the higher temperature regime. This is also because 220°C corresponds to a temperature frequently employed in home cooking, such as frying or pizza baking.

### Structure Assignment and Mass Spectrometry Data

**Figure 1:**
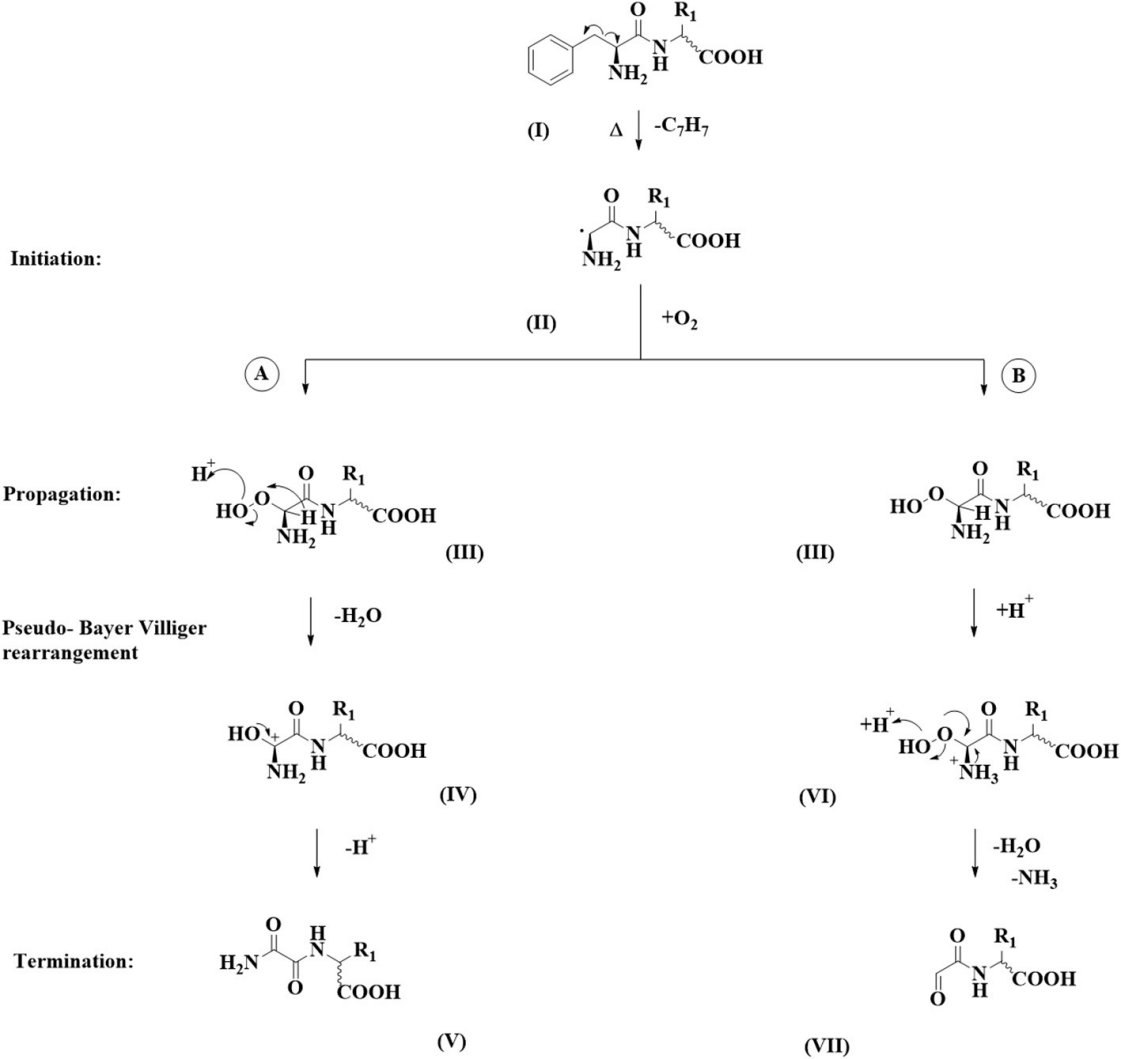
Dicarbonyl formation at the N-terminus of Phe *via* debenzylation. Two chemical pathways are followed: **(A)** a pseudo Baeyer-Villiger amide formation and **(B)** a peroxy-aminal hydrolysis.

In all cases around 20–30 different degradation products formed from a single pentapeptide precursor could be observed in the LC-MS chromatograms using our established chromatographic method. Next to previously reported decarboxylation products and tryptophan oxidation products (3) and peptides formed through amide bond hydrolysis, we could observe two to six compounds in selected chromatogram that, according to the UV trace, were devoid of the phenylalanine chromophore. These degradation products could be observed for FAFAW, FAKAW, FARAW, FAYAW, FAHAW, and WAKAF in higher abundance and for FATAW, FAQAW, and FADAW in lower abundance. All of these compounds showed a consistently reduced mass with molecular formulae assignment suggesting for all compounds a loss of a C_7_H_7_ moiety, hence suggesting a debenzylation step at the N-terminal Phe to give thermal degradation products with “pseudo_N-terminal” 1-aldehyde-2-amide and 1,2 diamide functionalities as shown in Figure 1.

Two custom pentapeptides have been selected to illustrate MS-based structure assignment: FARAW and FAHAW. Tables 1, 2 present the high-resolution masses of all their thermal degraded products, including the products of debenzylation highlighted in the tables (selected extracted ion chromatograms are given in the Supplementary Information; Supplementary Figures 1, 2).

**Table 1:** High resolution mass (UHPLC-Q-TOF-MS) and tandem MS data of thermal degraded products of FARAW and their parent ions [M + H]^+^.

**No**	**Mol. formula**	**Theor. *m/z* [M + H]^**+**^**	**Exp. *m/z* [M + H]^**+**^**	**Error (ppm)**	**RT (min)**	**MS/MS**
1	C_9_H_18_N_5_O_2_	228.1455	228.1451	1.7	3.1	169.0954 (100)
2	C_21_H_35_N_8_O_4_	463.2776	463.2779	−0.7	4.6	219.1071 (100); 212.1264 (34.1); 191.1187 (30.6)
3	C_12_H_17_N_2_O_3_	237.1234	237.1238	−1.7	10.2	212.9461 (100)
4	C_20_H_30_N_7_O_4_	432.2354	432.2353	0.2	16.7	246.1552 (100); 229.1252 (23.7)
5	C_14_H_18_N_3_O_3_	276.1343	276.1343	−0.1	20.8	188.0715 (100)
6	C_32_H_45_N_9_O_7_	333.6715	333.6712	1.1	21.5	211.1209 (100); 317.1929 (95.6); 221.0895 (91.4); 175.0847 (64.3)
7	**C**_**25**_**H**_**36**_**N**_**9**_**O**_**7**_	**574.2732**	**574.2733**	−0.1	25.9	388.1909 (100)
8	C_22_H_31_N_8_O_5_	487.2412	487.2407	0.9	29.9	215.0809 (100); 260.1045 (53.5); 399.1783 (42.1); 243.0702 (34.4)
9	C_25_H_35_N_8_O_7_	559.2623	559.2623	0.0	30.6	315.0932 (77.1); 287.1044 (36.7)
10	C_22_H_29_N_6_O_7_	489.2092	489.2085	1.4	31.2	-
11	C_33_H_44_N_9_O_7_	678.3358	678.3350	−0.1	34.5	492.25 (30.8); 247.1107 (26); 403.204 (25.7)
12	C_34_H_46_N_9_O_7_	692.3515	692.3514	0.1	35.1	-
13	C_34_H_47_N_10_O_7_	707.3552	707.3555	−0.5	35.8	689.3405 (56.9)
14	C_33_H_40_N_9_O_7_	674.3045	674.3046	−0.2	40.3	-

*Bold values correspond to the debenzylation products of the two thermally treated penatapeptides*.

**Table 2:** High resolution mass (UHPLC-Q-TOF-MS) and tandem MS data of thermal degraded products of FAHAW and their parent ions [M + H]^+^.

**No**	**Mol. formula**	**Theor. *m/z* [M + H]^**+**^**	**Exp. *m/z* [M + H]^**+**^**	**Error (ppm)**	**RT (min)**	**MS/MS**
1	C_9_H_13_N_4_O_2_	209.1033	209.1034	−0.7	2.7	181.1071 (82); 166.0593 (32.5); 192.0794 (22.8)
2	C_20_H_25_N_6_O_4_	413.1932	413.1930	0.3	16.9	209.1073 (100); 181.1061 (85.4); 188.0684 (43.3)
3	C_14_H_17_N_2_O_3_	261.1234	261.1236	−0.8	18.9	215.1293 (100); 320.9181 (43.8)
4	C_14_H_18_N_3_O_3_	276.1343	276.1342	0.4	21.1	188.0672 (100); 175.8593 (42.8)
5	**C**_**25**_**H**_**30**_**N**_**7**_**O**_**7**_	**540.2201**	**540.2203**	−0.2	30.5	452.1532 (100); 424.1754 (49.4); 209.1 (38.8); 226.1294 (36.2)
6	C_33_H_39_N_8_O_7_	659.2936	659.2944	−1.2	34.5	384.1660 (100); 455.2066 (91.5); 356.1781 (56.7); 427.2068 (32.6)
7	C_33_H_37_N_8_O_7_	657.2780	657.2779	0.1	37.5	382.1541 (100); 354.1616 (68.1); 453.19 (34.2)
8	C_33_H_35_N_8_O_7_	655.2623	655.2635	−1.9	39.7	352.1371 (100); 380.1409 (73.1)

*Bold values correspond to the debenzylation products of the two thermally treated penatapeptides*.

**Figure 2:**
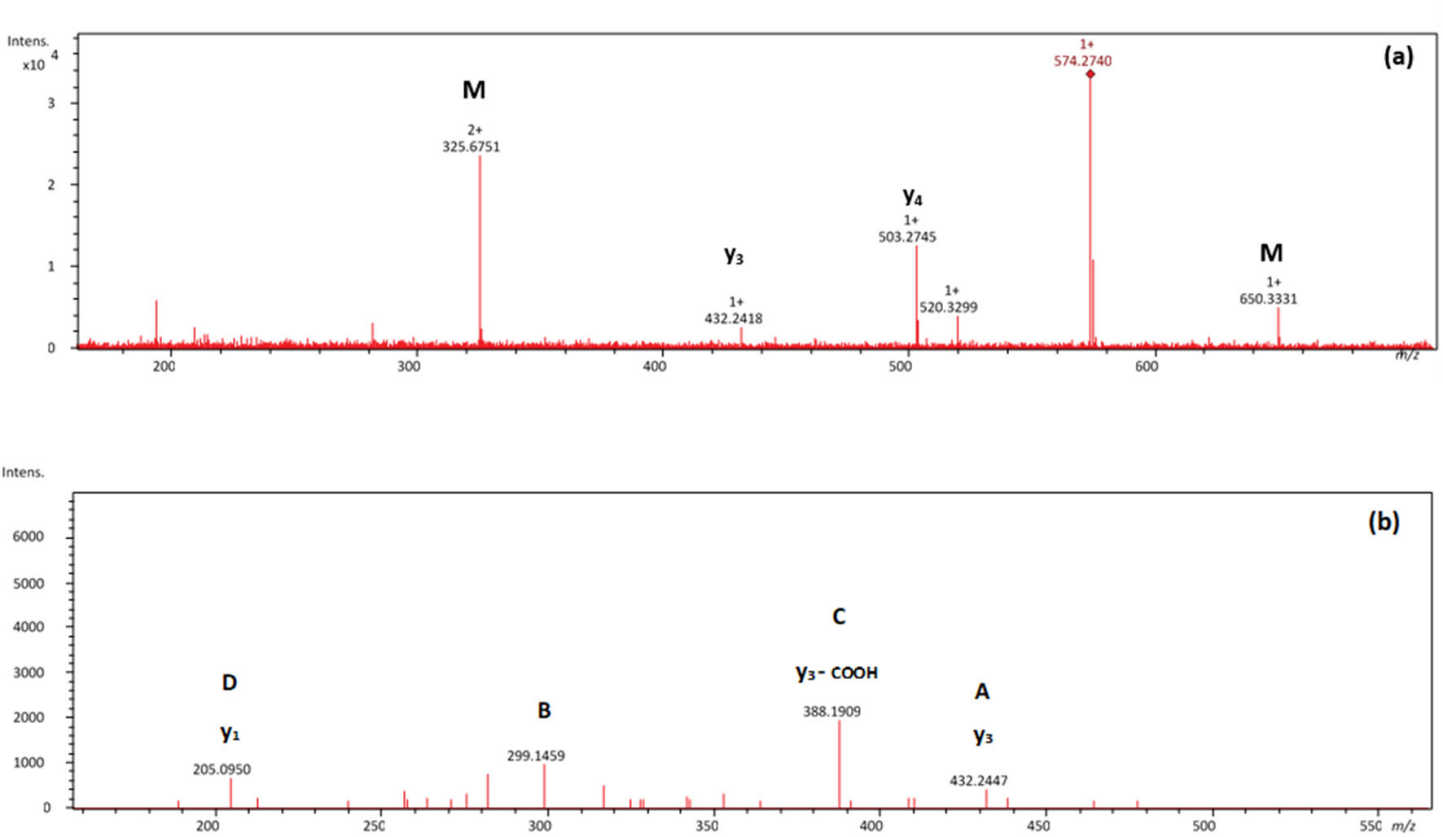
**(A)** Mass spectrum showing a NL of 76 Da of the thermally treated FARAW (*m/z* 574.2740) and **(B)** MS^2^ spectrum of the *m/z* 574.3 debenzylated product. Proposed fragmentation scheme is shown in Supplementary Figure 3.

**Figure 3:**
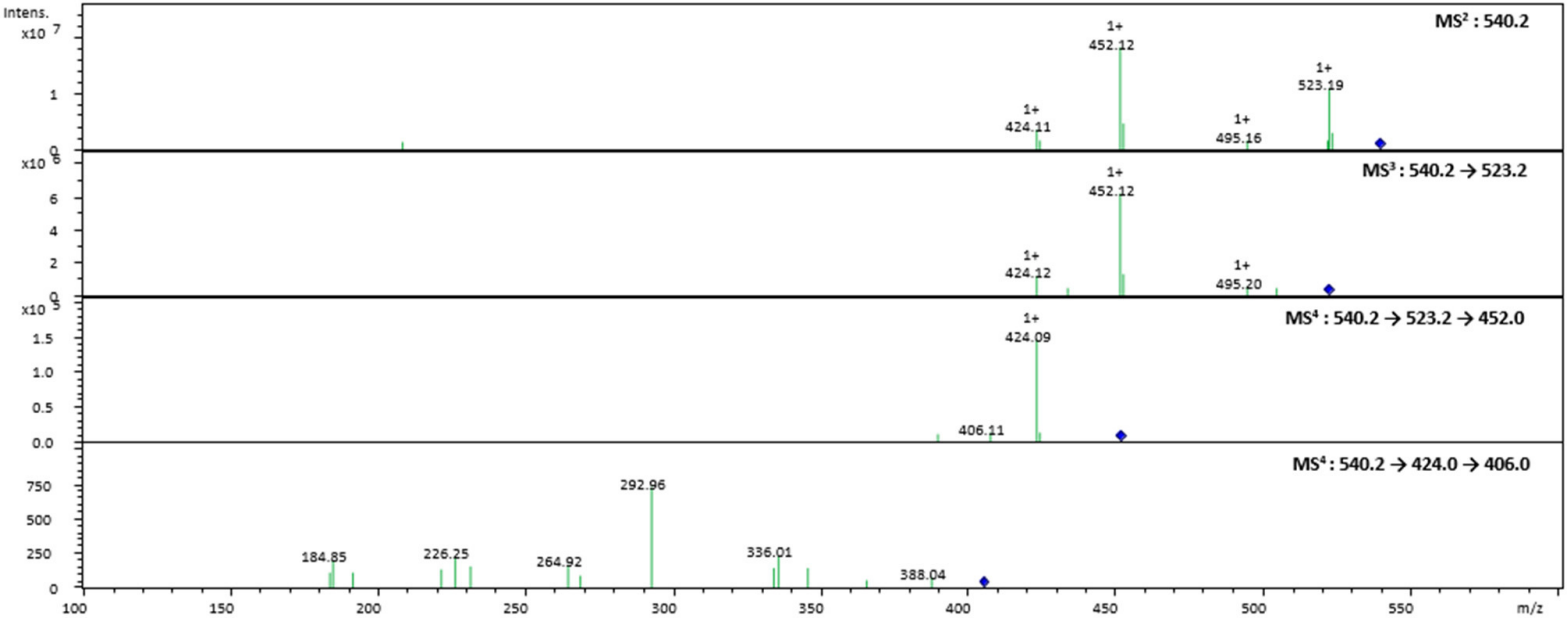
Tandem MS spectra of the pseudomolecular ion at *m/z* 540.2202 obtained by an ESI-IonTrap mass spectrometer.

Figures 2, 3 show selected full scan and tandem mass spectra of debenzylated products of FARAW and FAHAW, respectively. For FARAW the full scan spectrum shows the molecular ion at *m/z* 574.2740 accompanied by the y_4_ (at *m/z* 503.2745) and y_3_ ion (*m/z* 432.2418). The tandem MS with the M + H precursor ion at 574.2740 as precursor ion shows a y_1_, y_2_, and y_3_ fragment ion along a b_3_ fragment ion at *m/z* 299.1459. Using an ion trap mass spectrometer the MS^3^ fragment spectrum with 299.1 as precursor ions reveals fragment ions at *m/z* 282.1 and 255.1, corresponding to a neutral loss of NH_3_ and C=ONH_2_, respectively. These data clearly indicate debenzylation at the N-terminal phenylalanine with all expected Y ions present (y_1_ to y_4_). The only b ion observed reveals in MS^3^ the expected loss of the N-terminal primary amide moiety.

In the case of FAHAW, the thermal degraded product has an [M + H] pseudomolecular ion at *m/z* 540.2202 (Table 1). With this as the precursor ion, the MS^2^ spectrum shows a fragment ion at *m/z* 523.2 (Figure 3) corresponding to loss of OH at the C-terminus, *m/z* 495.2 corresponding to an additional loss of the formyl group at the pseudo-N-terminus, a base peak at *m/z* 452.1, corresponding to the z_4_ ion (loss of C_2_H_2_NO_2_), and finally at *m/z* 424.1 corresponding to the x_4_ ion (loss of C_4_H_6_NO_3_). The MS^3^ spectrum with the fragment ion at *m/z* 523 as precursor ion confirms the assignment with identical ions formed.

### Mechanism of Formation

The suggested products obviously arise through debenzylation at the N-terminus. For the pentapeptide with the inverse sequence WAKAF a loss of a methyleneindole moiety is observed corresponding to an analogous debenzylation at the N-terminus. The benzyl group is frequently used as a protecting group in organic chemistry synthesis, mainly due to its high stability and low reactivity (18). However, in many cases, under the presence of specific reagents or catalysts, its stability might render, leading to a C-C cleavage. Debenzylation of peptides is favored in the presence of formic acid, which behaves as a proton donor resulting in a catalytic transfer hydrogenation (19, 20). In our case, both elevated temperature, 180°C and 220°C, and the slightly acidic peptide environment seem to favor the debenzylation of phenylalanine mechanism occurring in more than half of the examined pentapeptides.

For product formation we suggest the following pathway, shown in Figure 1, supported by some additional experiments. Firstly, a homolytic C-C-bond cleavage leads to loss of a benzyl radical. The resulting secondary radical centered at the N-terminal α-carbon (**II)** is stabilized by resonance by the carbonyl amide C=O and by the lone pair on the primary amine. The observed homolytic C-C bond fission therefore is favored by the formation of two rather stable radical species. It needs to be noted that we did not find any such reaction precedent in the organic reaction literature. Stabilized amino acid α-carbon centered radicals have, however, been reported and used as intermediates in synthesis (21) or postulated as intermediates (22). In a second step we assume reaction with elemental oxygen, which, following a hydrogen radical abstraction, produces a peroxoaminal **(III)**. This intermediate can subsequently follow either one of two pathways: A or B. In pathway A we suggest protonation at the peroxy functionality followed by cleavage of the O-O bond, yielding a six valence electrons at oxygen intermediate. This is stabilized by migration of the α-carbon hydrogen toward the electron deficient oxygen. The resulting aminal cation **(IV)** yields the 1,2 diamide **(V)** following deprotonation. This reaction sequence is reminiscent of the Hock reaction or Bayer Villiger oxidation. In the molecular context the hydride group shows the highest migratory aptitude resulting in **(V)** as the expected product.

In pathway B peroxyaminal **(III)** follows a classical aminal hydrolysis pathway, yielding *via* intermediate **(VI)** the final 1-formyl-2amide **(VII)**.

Further evidence for this mechanism includes a negative control experiment carrying out the thermal degradation in the absence of oxygen. Here, no compounds of type **(V)** or **(VII)** could be detected. A thermal degradation in the presence of a pure oxygen atmosphere results in detection of the peroxyaminal intermediate at *m/z* 572.23 for FAHAW.

## Conclusion

In this contribution we show that N-terminal phenylalanine containing peptides degrade *via* a radical debenzylation pathway, forming two types of α-dicarbonyl peptides. This degradation pathway was found to occur in more than half of the examined pentapeptides. In a final step, dicarbonyl has been formed in the case of FARAW, FAHAW, and FGKGW.

The mechanism suggested involves formation of a peroxy species and is reminiscent of lipid peroxidation. Hence not only lipids but also peptides are subject to thermal peroxidation.

End products of the reaction are highly electrophilic. 1,2 dicarbonyl might serve as important building blocks in the formation of more complex Maillard reaction products, in particular what are referred to as Melanoidines (23). Glyoxal, the smallest dialdehyde, is known to be the end product in the glycation of proteins by glucose. In general, reactions between proteins and carbohydrates, known as Maillard reactions, may occur not only at elevated temperatures but also in body cells. The negative impact of advanced glycation end-products (AGEs) on health has been reported in literature, with glyoxal playing a significant role on the biological mechanism, which results in hyperglycemia (24, 25). The results obtained from this work, together with our knowledge about the health implications of dicarbonyls, raise new questions in the area of food processing, which should attract further interest in the future.

Protein and peptide degradation reactions in foods may lead to losses in food quality, but at the same time, may result in generation of degradation products of possible toxicological relevance. Chronic consumption of oxidized fat, for instance, is known to have a negative impact on human health. With this work, we would like to direct scientific interest and attention to protein and peptide degradation mechanisms during food heat treatment.

## Data Availability Statement

The original contributions generated for the study are included in the article/Supplementary Material, further inquiries can be directed to the corresponding author.

## Author Contributions

MB and NK jointly planned the research and wrote the manuscript. MB in addition carried out experimental work and data interpretation. All authors contributed to the article and approved the submitted version.

## Conflict of Interest

The authors declare that the research was conducted in the absence of any commercial or financial relationships that could be construed as a potential conflict of interest.

## Abbreviations

UHPLC: Ultra-High performance liquid chromatography
ESI: Electrospray ionization
Q-TOF: Quadrupole time of flight
MS: Mass spectrometry
FAFAW: phenylalanylalanylphenylalanylalanyltryptophane
FAKAW: phenylalanylalanyllysinylalanyltryptophane
FAYAW: phenylalanylalanyltyrosinylalanyltryptophane
FARAW: phenylalanylalanylargininylalanyltryptophane
FAHAW: phenylalanylalanylhistidinylalanyltryptophane
WAKAF: tryptophanylalanyllysinylalanylphenylalanine
FATAW: phenylalanylalanylthreonylalanyltryptophane
FADAW: phenylalanylalanylaspartylalanyltryptophane
FAQAW: phenylalanylalanylglutaminylalanyltryptophane.
