# Supplementary material for: “Thermal Peroxidation” of Dietary Pentapeptides Yields N-Terminal 1,2-Dicarbonyls
Source: Front Nutr. 2021 Jul 22;8:663233. doi: 10.3389/fnut.2021.663233 (PMC8339318; doi:10.3389/fnut.2021.663233)
Supplement: Supplementary file 1 [file Data_Sheet_1.pdf]

Supplementary Information

# Thermal degradation of pentapeptides yields N-terminal 1, 2-dicarbonyls by a debenzylation pathway

**Table S1: List of investigated pentapeptides.**

| No | Peptide sequence* | MW (Da) | Mol. Formula |
|---|---|---|---|
| 1 | FAFAW | 640.3009 | $C_{35}H_{40}N_6O_6$ |
| 2 | FAKAW | 621.3274 | $C_{32}H_{43}N_7O_6$ |
| 3 | FARAW | 649.3336 | $C_{32}H_{43}N_9O_6$ |
| 4 | FACAW | 596.2417 | $C_{29}H_{36}N_6O_6S_1$ |
| 5 | FASAW | 580.2645 | $C_{29}H_{36}N_6O_7$ |
| 6 | FATAW | 594.2801 | $C_{30}H_{38}N_6O_7$ |
| 7 | FAYAW | 656.2958 | $C_{35}H_{40}N_6O_7$ |
| 8 | FAQAW | 621.2910 | $C_{31}H_{39}N_7O_7$ |
| 9 | FADAW | 608.2594 | $C_{30}H_{36}N_6O_8$ |
| 10 | FAEAW | 622.2751 | $C_{31}H_{38}N_6O_8$ |
| 11 | FAHAW | 630.2914 | $C_{32}H_{38}N_8O_6$ |
| 12 | FPKAW | 647.3431 | $C_{34}H_{45}N_7O_6$ |
| 13 | FAKPW | 647.3431 | $C_{34}H_{45}N_7O_6$ |
| 14 | WAKAF | 621.3274 | $C_{32}H_{43}N_7O_6$ |
| 15 | FGKGW | 593.2961 | $C_{30}H_{39}N_7O_6$ |

* Each peptide sequence is represented by the single letter codes for each of the amino acids.

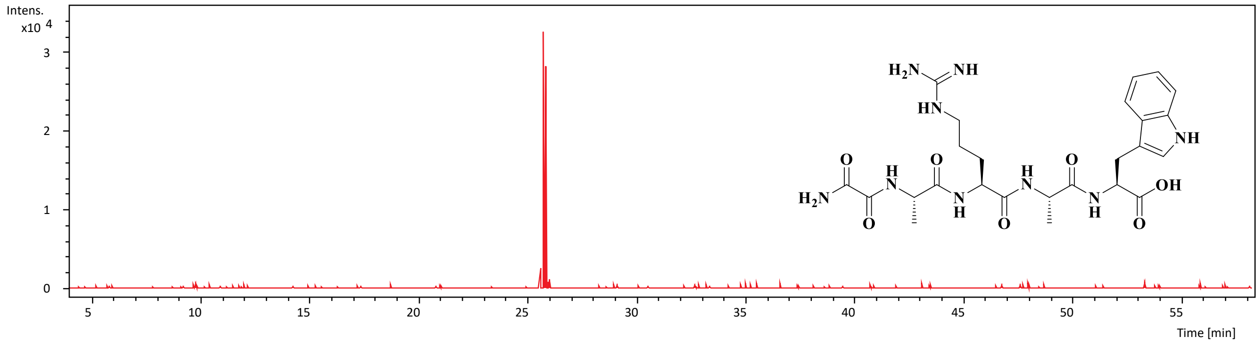


**Figure S1:** Extracted ion chromatogram (EIC) at *m/z* 574.3 showing the debenzylated and oxidised FARAW at the N-terminus at RT: 25.9 min.

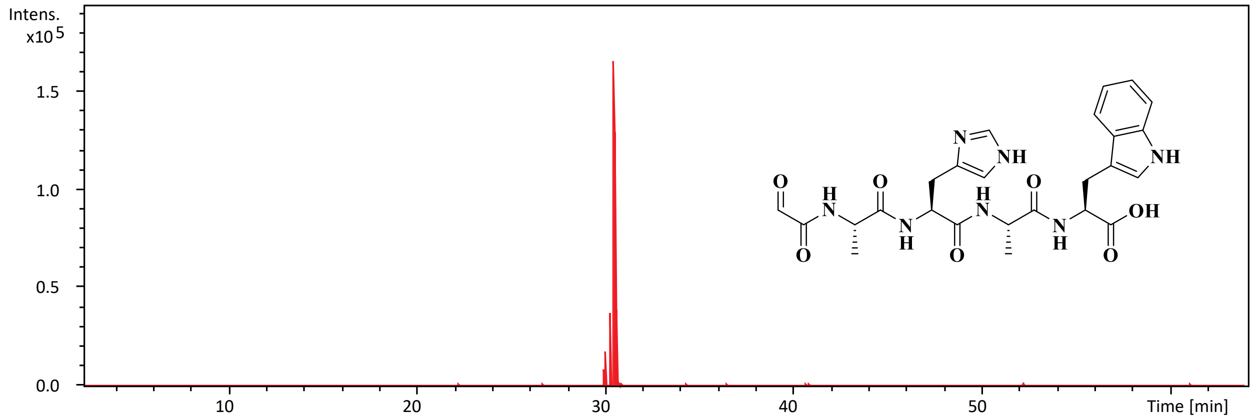


**Figure S2:** Extracted ion chromatogram (EIC) at *m/z* 540.3 showing the thermal modification of FAHAW forming a dicarbonyl derivative.

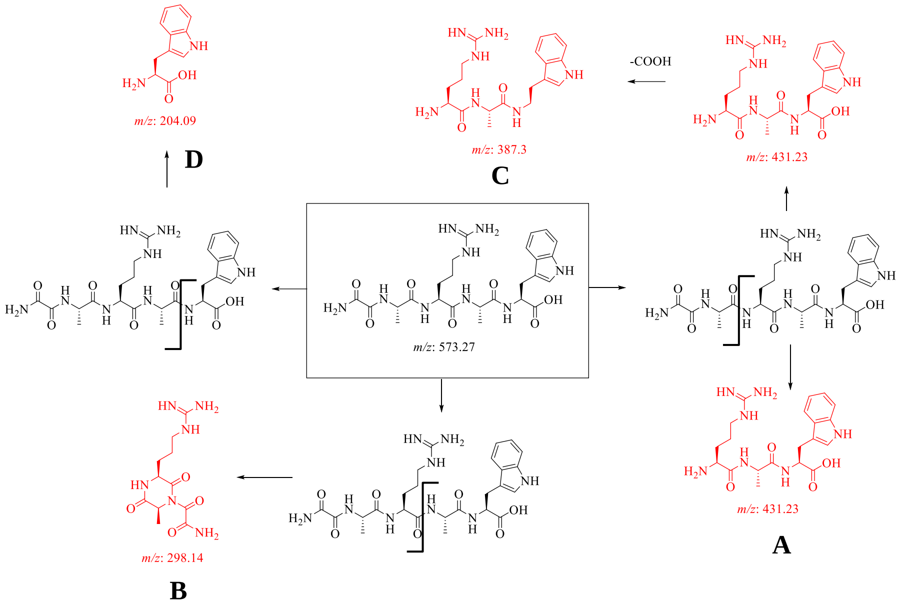


**Figure S3:** Proposed fragmentation scheme of debenzylated and oxidised FARAW.
